# Biological and functional evaluation of a novel pyrolytic carbon implant for the treatment of focal osteochondral defects in the medial femoral condyle: assessment in a canine model

**DOI:** 10.1186/s13018-016-0488-5

**Published:** 2016-12-01

**Authors:** Samantha L. Salkeld, Laura P. Patron, Joan C. Lien, Stephen D. Cook, Deryk G. Jones

**Affiliations:** 1 Fellowship of Orthopaedic Researchers, 320 Metairie Hammond Highway, Suite 406, Metairie, LA 70005 USA; 2 Department of Sports Medicine and Cartilage Restoration, Ochsner Sports Medicine Institute, Jefferson, LA USA

**Keywords:** Pyrolytic carbon, Hemiarthroplasty, Osteochondral defect, Medial femoral condyle, Cartilage wear, Animal model

## Abstract

**Background:**

Osteochondral defects continue to be a clinical treatment challenge, and when left untreated, may cause pain and functional impairment. Pyrolytic carbon is a unique isotropic biomaterial used in heart valve and small joint replacements due to its excellent wear properties and biocompatibility with bone and articular cartilage. Therefore, a proposed solution is to utilize a focal pyrolytic carbon hemiarthroplasty implant as an alternative resurfacing treatment strategy for isolated cartilage lesions.

**Methods:**

A canine model (*n* = 9) was used to evaluate the in vivo histologic response and function of a pyrolytic carbon implant replacing a full-thickness osteochondral defect in the medial femoral condyle (MFC) of the knee. The gross appearance and histologic results were compared to an identical cobalt-chromium (Co-Cr) alloy implant placed in a defect in the contralateral MFC and evaluated up to 52 weeks.

**Results:**

Extensive bone incorporation to the stem portion was observed for both implant types. The total mean histologic score for the cartilage of the MFC surrounding the pyrolytic carbon implants was significantly improved compared to that of the Co-Cr alloy implants at all evaluation periods (*p* < 0.05). Histologic grading and gross observations at 52 weeks for pyrolytic carbon implants were similar to those of Co-Cr alloy implants at 24 weeks. At 24 weeks, the mean total histologic score for Co-Cr alloy implants was 11.6 ± 0.7 (0–16 range point; 16 = normal appearance), while at 52 weeks, the mean total score for the pyrolytic carbon implants was 11.7 ± 1.3. Mean total histologic score of opposing medial tibia cartilage for the pyrolytic carbon implants was superior to that of the Co-Cr alloy group at all evaluation periods and significantly improved over the Co-Cr alloy implant group at 24 weeks (*p* = 0.001) and 52 weeks (*p* < 0.001).

**Conclusions:**

Use of a pyrolytic carbon implant for reconstruction of a focal cartilage defect demonstrated effective implant fixation and superior in vivo response compared to an identical Co-Cr alloy implant.

## Background

Osteochondral defects of the medial femoral condyle negatively impact functionality of the knee. Symptomatic lesions are generally painful and disabling, and can progress to osteoarthritis when left untreated. Not only is the repair tissue in a focal chondral or osteochondral defect inferior to normal articular cartilage but the presence of defects can also damage the opposing meniscus and tibial cartilage surface [1, 2].

Currently, there are several treatments available, which depending on the goal of the intervention, may be directed at relieving symptoms, regenerating tissue, or repairing the defect. Arthroscopic debridement and lavage alleviate symptoms but the long-term result for the lesion is poor [1, 3–6]. Abrasion chondroplasty, subchondral drilling, and microfracture have been shown to regenerate tissue with acceptable results, but the resulting fibrocartilage lacks the strength, organization, and biomechanics of normal articular cartilage [1, 7, 8]. Autologous chondrocyte implantation has produced good to excellent outcomes, but the multi-step procedure involves extensive rehabilitation, may not be appropriate for older or heavier patients, and also carries a risk of donor site morbidity [1, 9, 10]. In severe cases of osteoarthritis or in cases where other regenerative or revision techniques have failed, a total knee arthroplasty (TKA) is the most effective solution. However, total knee arthroplasty is a final resort for many patients due to likelihood of revision surgeries (usually within 15–20 years) and associated morbidity throughout the patient’s lifetime. For younger patients with TKAs, revision surgeries are more likely due to increased activity of younger patients which leads to greater implant wear and reduced implant lifespan [11–14].

A proposed solution is a focal hemiarthroplasty implant, intended for use as an alternative resurfacing treatment strategy for isolated lesions in patients who are too young for TKAs [1] or not good candidates for regenerative procedures. Focal hemiarthroplasty devices using pyrolytic carbon [15], metal alloys [15, 16], polymer composites [17], and ceramic composites [18] have been investigated for a number of anatomical locations with varying results. Several previous studies have shown that even when the shape and design of the implant closely match the surface, the implant material properties may be detrimental to the opposing articular cartilage in the long-term due to differences in elastic modulus [15, 19, 20]. A mismatch in elastic properties contributes to higher stresses around the implant-bone interface, which leads to bone loss due to stress shielding, followed by bone resorption and implant loosening [21, 22].

Wear of articulating surfaces can also be a major limiting factor in arthroplasty and hemiarthroplasty. In the natural joint, surface-active phospholipids (SAPL) and their major component, dipalmitoylphosphatidylcholine (DPPC), have been found to possess remarkable capabilities in reducing friction and are excellent anti-wear agents even under high loads [23, 24]. Thus, researchers have identified that SAPL acts as the active boundary lubricant in both natural and artificial joints. A deficiency in SAPL, as in osteoarthritis, results in sticking of the articulating surfaces [25]. Purbach et al*.* [26] was the first to report on the presence of SAPL on artificial joints. Analysis of total hip replacement prostheses showed that sufficient SAPL was present to form oligolamellar layers on the bearing surfaces, suggesting that this layered structure of phospholipids acts similar to lamellated solid lubricants and protects the surface from wear.

Pyrolytic carbon has been shown to have superior qualities for use in partial and total joint replacements due, in part, to its similar elastic modulus to bone, which reduces stress shielding [27]. Pyrolytic carbon also has high strength, high fatigue resistance, and high wear resistance even when subjected to cyclic loads [28–30]. Pyrolytic carbon has been shown to be biocompatible with both hard and soft tissues, which is important for minimizing host inflammatory response to an implanted medical device [29, 31–33]. Studies have also shown that DPPC has a high affinity for pyrolytic carbon, indicating that SAPL will likely do the same [34, 35], thus effectively lubricating and reducing the friction between cartilage articulating against a pyrolytic carbon implant surface.

Experimental studies have been conducted using pyrolytic carbon as a hemiarthroplasty material. Cook et al*.* [28] evaluated degeneration of acetabular cartilage following implantation of proximal femoral hemiarthroplasties in dogs. The articulating surface of the femoral component was composed of either pyrolytic carbon, cobalt-chromium-molybdenum (Co-Cr-Mo) alloy, or titanium alloy. Results indicated that native acetabulum in articulation with pyrolytic carbon implants experienced a lesser degree of joint degeneration and there was significantly greater probability of cartilage survival with articulation against the pyrolytic carbon implant compared to the metal alloy implants. In an osteoarthritis model, Kawalec et al. [15] examined the histologic response to pyrolytic carbon and cobalt-chromium (Co-Cr) alloy hemiarthroplasty implants in the lateral femoral condyle of the knee in beagle dogs compared to an untreated cartilage control group. To simulate degeneration and loss of cartilage associated with osteoarthritis, Kawalec et al. created an osteochondral defect on the opposing lateral surface of the tibial plateau that articulated with a pyrolytic carbon device, a similar Co-Cr device, or the native condyle cartilage. Results showed no evidence of adverse inflammatory response, and fibrocartilage regeneration in 86% of knees treated with the carbon implants but only in 25% of metal implants. Improved fibrocartilage regeneration was attributed to the more “normal” biomechanical condition of the bone-carbon articulation than the bone-metal articulation.

The objective of this study was to use a canine model to evaluate the in vivo histologic response and function of a pyrolytic carbon implant in the medial femoral condyle of the knee and to compare the results to those obtained using an identical Co-Cr alloy implant.

## Methods

### Test devices

**Fig. 1:**
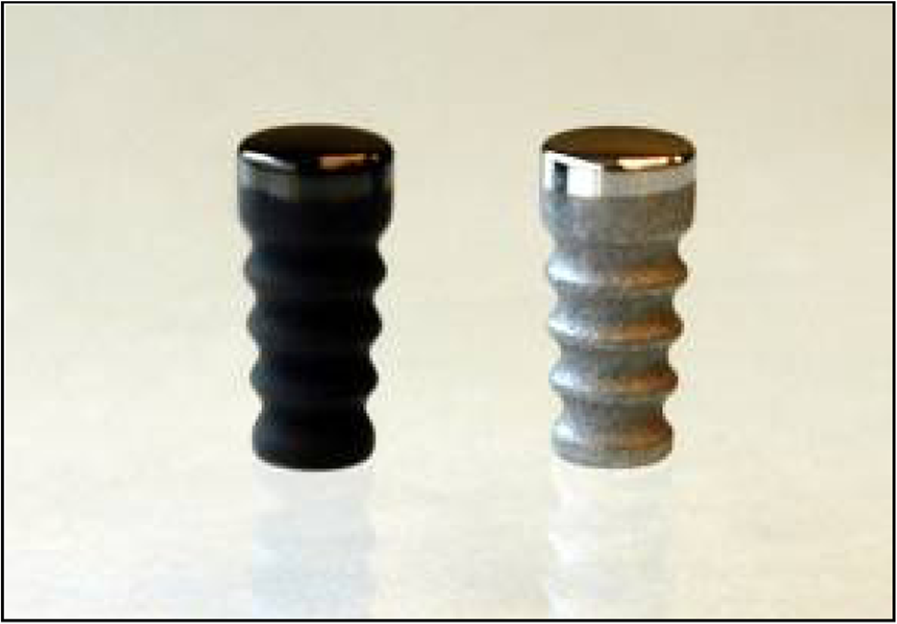
Pyrolytic carbon (*left*) and Co-Cr alloy (*right*) implant devices

The device used in this study was a weight-bearing, cementless, single-component articular implant fabricated from either pyrolytic carbon or Co-Cr alloy and designed for implantation in the medial femoral condyle of the study animals (Fig. 1). The devices had an articulating surface with a 6-mm diameter and 11-mm radius of curvature. The stem was tapered with a series of four grooves, and the total height of the device was 12.35 mm. The pyrolytic carbon implants were machined from graphite with a pyrolytic carbon coating applied in a fluidized bed reactor. The implant’s stem surface had the as-deposited pyrolytic carbon material, while the articulating surfaces were polished to less than 2 μin. The articulating surfaces of the Co-Cr alloy implants were also polished to less than 2 μin. and the stem portion had a grit-blasted surface. The choice of a Co-Cr alloy analogous implant to the pyrocarbon implant was made based on currently available similarly functioning clinical implants such as the Co-Cr alloy HemiCAP device (Arthrosurface Inc., Franklin, MA) and the characterization of Co-Cr as a control articular implant in the literature in this application [15, 16] and in a similar articular joint application [28].

### Experimental design

Nine adult male beagle dogs, 1.6 ± 0.1 years of age, were used in the study (12.8 ± 2.2 kg). The study was undertaken after receiving approval from the In-Life Facility’s IACUC committee for all animal care and procedures. The animals were assigned to the following evaluation periods: 12 weeks post-surgery (*n* = 3), 24 weeks post-surgery (*n* = 3), and 52 weeks post-surgery (*n* = 3).

### Surgical procedure and radiographic evaluation

**Fig. 2:**
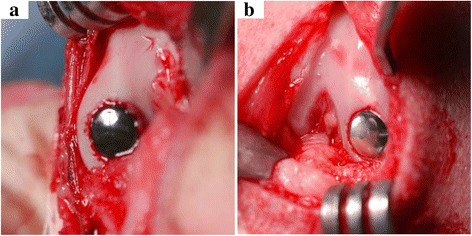
Inter-operative photograph of placement of pyrolytic carbon (**a**) and Co-Cr (**b**) implants in the medial condyle

All animals underwent bilateral implantation of the devices in the medial femoral condyle (pyrolytic carbon in one knee and Co-Cr alloy in the other, Fig. 2). Under sterile conditions, the medial capsule of the knee joint was approached through a median parapatellar incision. The medial retinaculum was partially released to dislocate the patella and the knee flexed to expose the medial femoral condyle. After exposure, a tapered defect representative of a grade IV cartilage lesion was produced using a series of custom guides and drills. The implant was then seated into the surgically created osteochondral defect site, using custom insertion instruments to ensure device alignment and surface continuity of the implant and articular surface. Anterior-posterior (AP) and lateral radiographs of the joint were taken immediately before and after surgery and at 6, 12, 24, and 52 weeks postoperative. Animals received tramadol (Ultram, 25 mg/dog, BID, oral) from day 1 to 5 (day 0 = surgical day) for management of postoperative pain. Animals received buprenorphine (0.01 mg/kg, intramuscular or subcutaneous (SC)) as deemed appropriate by the attending veterinarian to control moderate to severe pain. Acepromazine (0.2 mg/kg, SC) was administered as needed to minimize distress and improve anesthetic recovery.

### Lameness evaluation

A single, blinded observer recorded the extent of lameness to each knee joint using a numerical grading scale modified from Paradis et al. [36]. A lameness score of 0 was given if the animal could stand, walk, and trot normally; 1, if they stood normally and had a slight algetic gait; 2, if they stood abnormally and had a slight algetic gait; 3, if they stood normally and had an evident algetic gait; and 4, if they stood abnormally and had an evident algetic gait. Evaluations were performed once every 3 days during the first three postoperative weeks and then once at each radiograph interval (6, 12, 24, and 52 weeks postoperative).

### Gross necropsy

Immediately following sacrifice, knee joints were harvested and contact radiographs taken. Harvested joints were grossly assessed for observations of the articular surfaces and surrounding cartilage, meniscus, and synovial joint tissues, as well as any discoloration or degenerative changes of the surrounding cartilage and discoloration of the synovial fluid.

### Histology of medial femoral condyle cartilage and observation of bone-implant interface

Following gross evaluation, specimens for histology were fixed in 10% formalin and dehydrated in graduated ethyl alcohol solutions. Specimens were then embedded in methyl methacrylate monomer, allowed to polymerize, and sectioned in the sagittal plane central to the implant’s long axis. Three centermost ground sections relative to the condyles were obtained, mounted, and ground/polished to approximately 50 μm thick. Sections were stained with Paragon. Microradiographs of the final sections were made using high-resolution Faxitron film (Industrex, Carestream). Undecalcified histologic sections were assessed using a semi-quantitative grading scale (Table 1), adapted from histologic assessments made by Kirker-Head et al. [16]. The percentages of bone contact, soft tissue contact, and void space contact with the implant were measured via digital images taken at ×2 (Nikon DXM1200) and image analysis software (Image-Pro Plus, Media Cybernetics).

**Table 1:** Histology grading scale (modified from Kirker-Head et al. [16]) used in evaluation of medial femoral condyle cartilage

Feature	Score
I. Adverse inflammatory response (0–3)	
None Slight Moderate Severe	3 2 1 0
II. Cartilage matrix flow over implant (0–3)	
None Slight, one side Slight, both sides Significant	3 2 1 0
III. Implant-bone apposition (0–3)	
Extensive Moderate Some None	3 2 1 0
IV. Medial femoral condyle cartilage-implant interface (0–3)	
Intact, normal Intact, cellular changes Moderate disruption Severe disruption	3 2 1 0
V. Medial femoral condyle cartilage (0–3)	
Normal Some changes, one side Some changes, both sides Significant degeneration	3 2 1 0
Total score (0–15)	

### Tibial articular cartilage evaluation

Medial and lateral tibial plateaus with intact menisci were collected and processed using decalcified paraffin-embedded techniques. Each specimen was sectioned into medial, central, and lateral blocks. From each block, three serial sections (5 μm thick) were obtained and stained with H&E, toluidine blue, and safranin O/fast green. Medial sections were examined using a modified ICRS-Histological Visual Scale [37], which was modified to reflect changes to existing hyaline cartilage by the presence of an articulating implant (Table 2). Lateral sections were observed as comparative controls for cartilage and staining.

**Table 2:** Histology grading scale (modified from Mainil-Varlet et al. [37]) used in evaluation of medial menisco-tibial articulating tissues

Feature	Score
I. Surface (0–3)	
Smooth and continuous Slight irregularities Moderate irregularities Discontinuous and irregular	3 2 1 0
II. Matrix (0–3)	
Hyaline Mixture of hyaline and fibrocartilage Fibrocartilage Fibrous tissue	3 2 1 0
III. Cell distribution (0–3)	
Columnar Mixed columnar and clusters Clusters Individual cells, disorganized	3 2 1 0
IV. Cell population viability (0–3)	
Intact–normal Intact–cellular changes Moderate disruption Severe disruption	3 2 1 0
V. Cartilage thinning/erosion, loss of stain (0–3)	
Normal Loss of staining Thinning Severe erosion	3 2 1 0
VI. Subchondral bone (0–3)	
Normal Active remodeling Severe bony changes Bone necrosis and/or granulation tissue	3 2 1 0
VII. Mineralization (0–3)	
Normal Slight changes Moderate changes Abnormal, inappropriate flocculation	3 2 1 0
VIII. Meniscus (0–3)	
Normal Slight changes Moderate disruption Severe disruption	3 2 1 0
Total score (0–24)	

### Statistical methods

The pyrolytic carbon and Co-Cr alloy devices were evaluated as pair-wise, within-animal comparisons. Descriptive statistics of histologic data were calculated. All quantitative data were initially screened for statistical outliers, defined as data points with z-scores of 3.0 or greater. Analysis of variance (ANOVA) was used to determine the effect of the experimental group (pyrolytic carbon and Co-Cr alloy) and postoperative time (12, 24, and 52 weeks). Non-parametric ANOVA was used to determine the effect of the experimental group and postoperative time on the histologic scores (Kruskal-Wallis test [38]). Significance (*) was defined as *p* ≤ 0.05. Data are presented as mean ± standard deviation.

## Results

### Surgical procedure, lameness evaluation, and radiographic evaluation

All animals tolerated the procedures well, had uneventful recoveries, and remained in good health until time of sacrifice. Within 4 weeks postoperative, all animals were ambulatory without evidence of lameness or impairment, and had normal stance bilaterally without evidence of non-weight bearing or pain during walking. Lameness scores did not exceed a grade of 2 (generally noted bilaterally), and evidence of slight lameness did not persist beyond week 3. One animal had three occurrences of right hindlimb lameness (Co-Cr alloy implant) at 14, 17, and 25 days postoperative, in which the contralateral hindlimb (pyrolytic carbon implant) was normal.

**Fig. 3:**
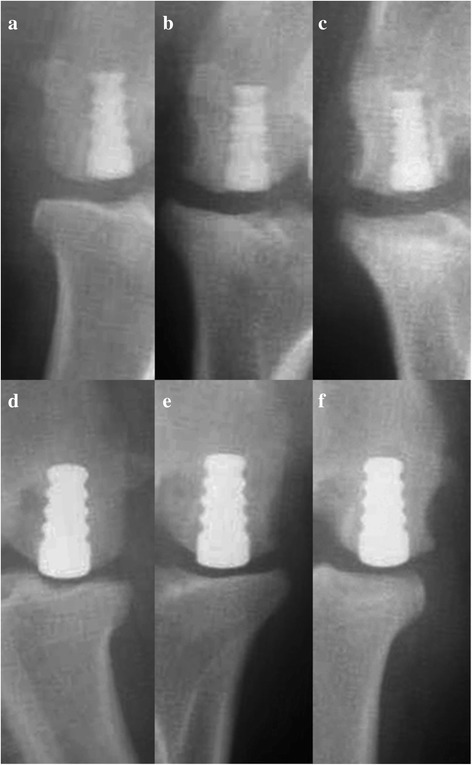
Postoperative radiographs: pyrolytic carbon (*top*) and Co-Cr (*bottom*) implants at 12 weeks (**a**, **d**), 24 weeks (**b**, **e**), and 52 weeks (**c**, **f**)

Review of postoperative radiographs showed adequate placement of all implants (Fig. 3). There was no radiographic evidence of loosening of the implants during the course of this study. Radiolucency around the pyrolytic carbon implant was the result of the radiolucent pyrolytic carbon coating thickness, and this was accounted for in the observations.

### Gross necropsy

**Fig. 4:**
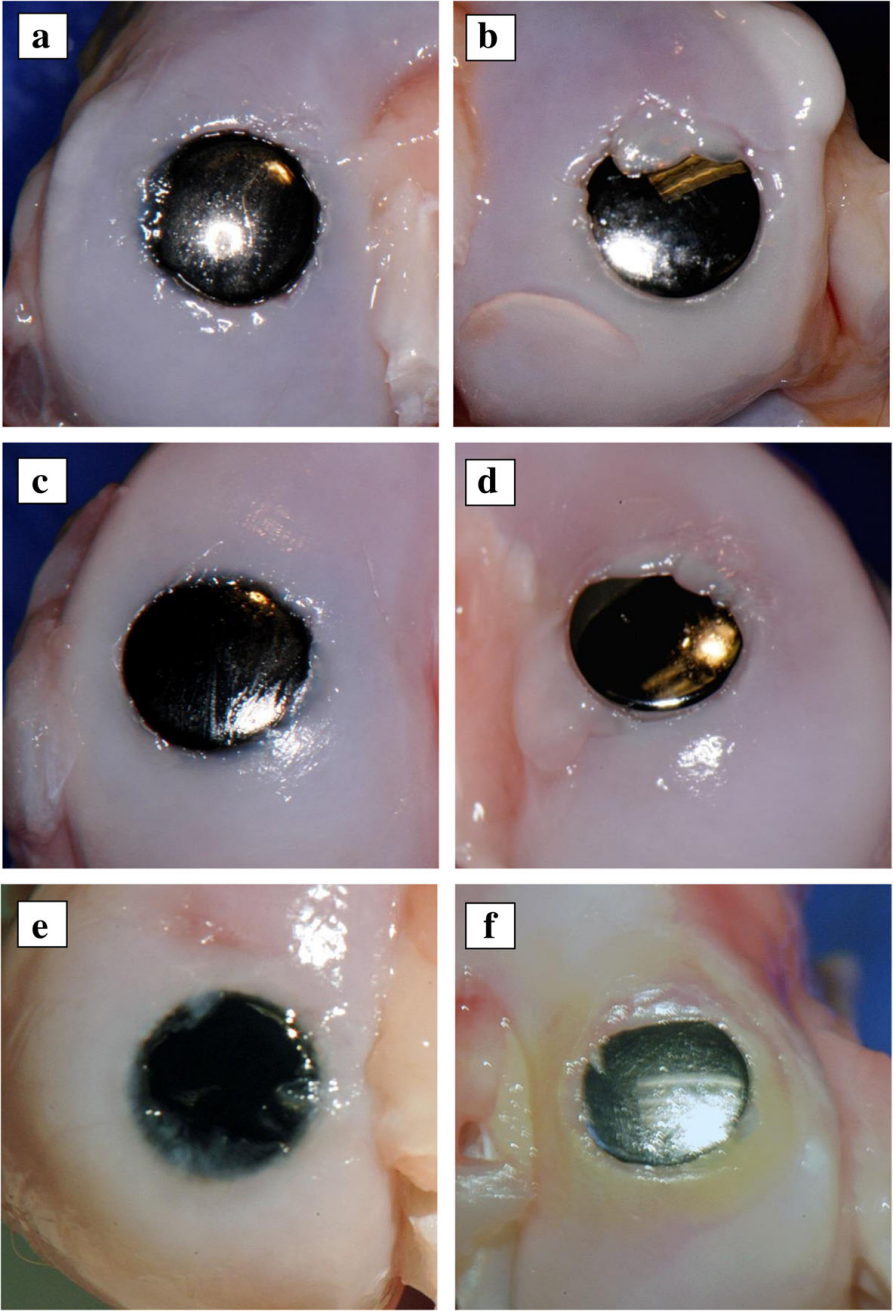
Gross appearance of pyrolytic carbon (*left*) and Co-Cr alloy (*right*) implants at 12 weeks (**a**, **b**), 24 weeks (**c**, **d**), and 52 weeks (**e**, **f**)

At 12 weeks, gross observations (Fig. 4) of the pyrolytic carbon implanted knees showed no evidence of articular cartilage damage or color change; no joint capsule hypertrophy, osteophyte formation, or meniscal changes related to the implant; and normal appearance of the synovial fluid. Gross observations of the Co-Cr alloy implanted knees showed no evidence of articular cartilage damage, one instance of opaque articular cartilage, two instances of joint capsule hypertrophy, one instance of osteophyte formation, and one instance of mild degeneration/streaking and thin, translucent appearance of the medial meniscus.

At 24 weeks, observation of the pyrolytic carbon implanted knees revealed one knee with moderate degeneration of the cartilage and minimal changes to the medial tibial plateau. For the Co-Cr alloy implanted knees, two had minimal fibrillation/degeneration of the cartilage on both the medial femoral condyle and medial tibial plateau surfaces, one with slight opacity changes in the articular cartilage, one with a thickened joint capsule, one osteophyte presence, one medial meniscus with mild degeneration/streaking, and no finding related to the gross appearance of the ligaments.

At 52 weeks, two of the pyrolytic carbon implanted knees had moderate degenerative changes of the cartilage on the medial femoral condyle with no changes on the medial tibial plateau surfaces and both had slight opacity changes to the articular cartilage. An osteophyte was present on one knee with mild streaking on the rim of one medial meniscus. All ligaments appeared normal. For the Co-Cr alloy implanted knees, one knee had moderate fibrillation/degeneration of the cartilage on both the medial femoral condyle and the medial tibial plateau surfaces and the articular cartilage was discolored. One knee had a thickened joint capsule with hypertrophy of the lateral collateral ligaments, two knees had condylar osteophytes, and one knee had a medial meniscus with evidence of moderate degeneration and a thinned, translucent appearance.

### Histology of medial femoral condyle cartilage and observation of bone-implant interface

**Fig. 5:**
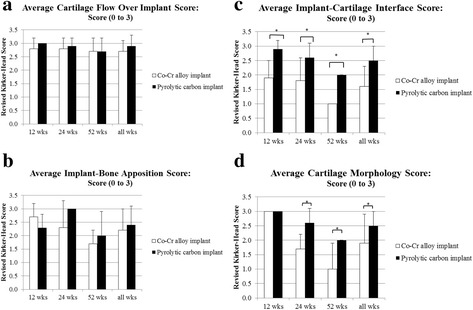
MFC histologic scores, means. **a** Cartilage flow over implant. **b** Implant-bone apposition. **c** Implant-cartilage interface. **d** Cartilage morphology. *p < 0.05

**Fig. 6:**
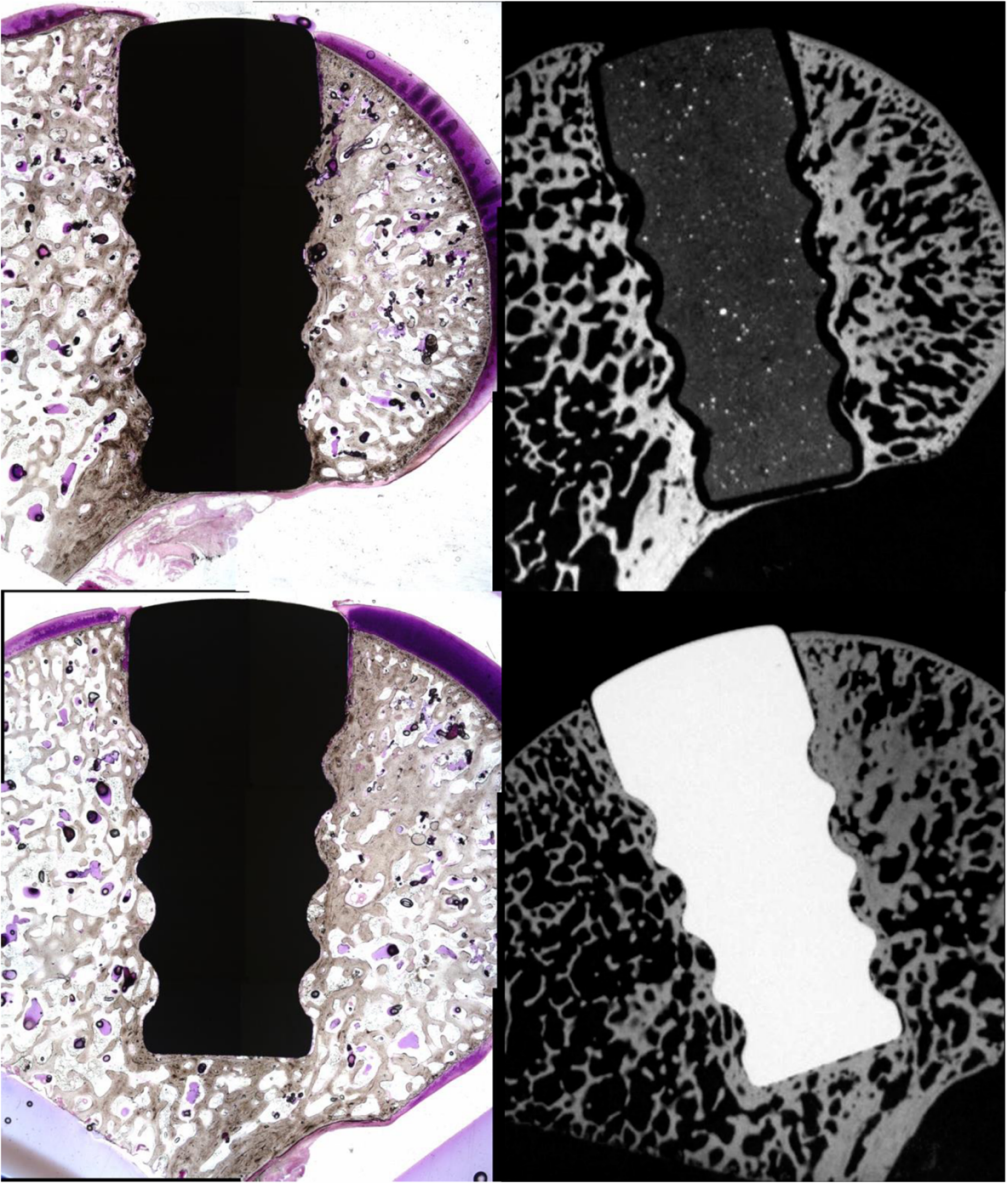
52-week histologic sections: microradiographs of pyrolytic carbon (*top*) and Co-Cr alloy (*bottom*) implants. Note radiolucent pyrolytic carbon coating surrounding radio-dense substrate (×2)

**Fig. 7:**
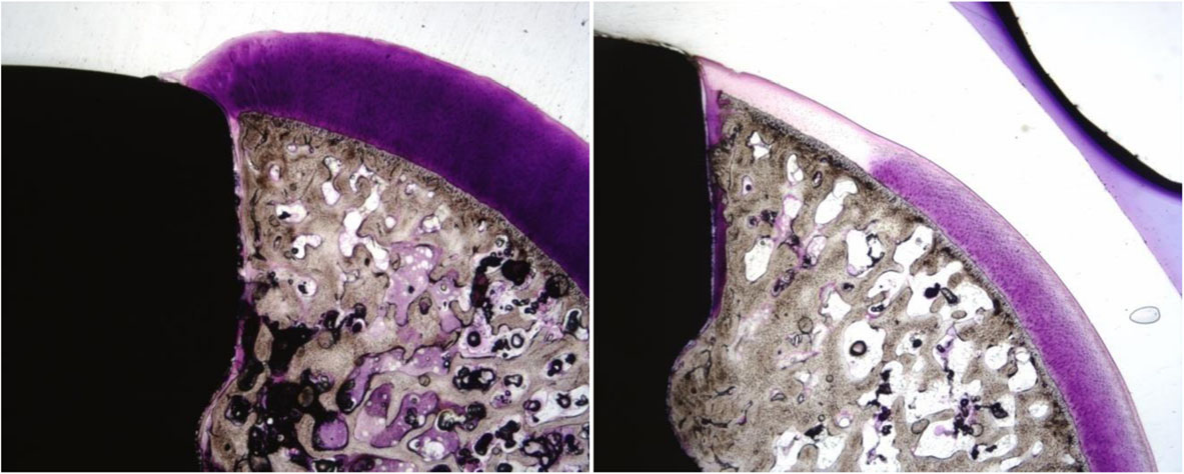
52-week sections of a pyrolytic carbon (*left*) and Co-Cr alloy (*right*) implant. Toluidine blue, basic fuchsin, magnification: ×2

**Fig. 8:**
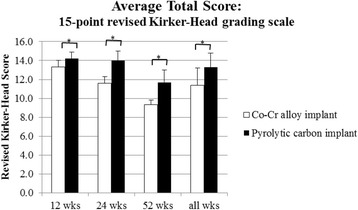
Mean total histologic scores for MFC cartilage. Significance, *p* < 0.05*

Histologic grading of the medial femoral condyle tissues showed that no significant inflammatory response was observed to either implant material at any evaluation period. The scores for the amount of cartilage flow over the implant (Fig. 5a) and the degree of implant-bone apposition (Fig. 5b) showed no significant differences between the two groups at any time point (Fig. 6). The mean implant-cartilage interface score for the pyrolytic carbon group was significantly improved compared to that of the Co-Cr alloy group at all time points (*p* < 0.05) (Fig. 5c). Histologic sections at 52 weeks postoperative demonstrated excellent maintenance of the host cartilage-implant interface with minimal cellular and mechanical changes present for the pyrolytic carbon implant, while significant cellular changes with loss of matrix staining was observed for the Co-Cr alloy implant at and near the host cartilage-implant interface (Fig. 7). The mean cartilage morphology score for the pyrolytic carbon group was significantly improved compared to that of the Co-Cr alloy group at 24 and 52 weeks (Fig. 5d). Overall, the mean total score of the pyrolytic carbon group was significantly improved over that of the Co-Cr alloy group at all evaluation periods (*p* < 0.05) (Fig. 8). Histologic grading and observations at 52 weeks for pyrolytic carbon implants were similar to those of Co-Cr alloy implants at 24 weeks. At 24 weeks, the mean total score for Co-Cr alloy implants was 11.6 ± 0.7, while at 52 weeks, the mean total score for the pyrolytic carbon implants was 11.7 ± 1.3.

There were no significant differences (paired *t* test, *p* > 0.05) observed between the two test groups regarding the quantitative measurements of mean percent bone contact (*p* = 0.617), percent soft tissue contact (*p* = 0.470), and percent void space at the implant interface (*p* = 0.964) (Table 3).

**Table 3:** Quantitative measurements of histologic parameters at the bone-implant interface

	Bone contact (%)	Soft tissue contact (%)	Void space (%)
Co-Cr alloy (*n* = 9)	34.5 ± 15.9	13.7 ± 12.1	51.8 ± 9.2
12 weeks (*n* = 3) 24 weeks (*n* = 3) 52 weeks (*n* = 3)	27.8 ± 5.9 35.8 ± 12.2 39.9 ± 26.8	12.7 ± 8.0 15.2 ± 16.6 13.3 ± 15.6	59.5 ± 4.7 49.0 ± 6.8 46.9 ± 11.4
Pyrocarbon (*n* = 9)	30.2 ± 20.4	17.4 ± 11.6	52.4 ± 19.2
12 weeks (*n* = 3) 24 weeks (*n* = 3) 52 weeks (*n* = 3)	20.8 ± 6.3 45.5 ± 11.3 24.5 ± 31.3	20.6 ± 16.3 17.1 ± 7.8 14.4 ± 19.5	58.5 ± 5.3 37.5 ± 7.5 61.3 ± 21.0
*p* value (paired *t* test)	0.617	0.470	0.964

### Tibial articular cartilage evaluation

**Fig. 9:**
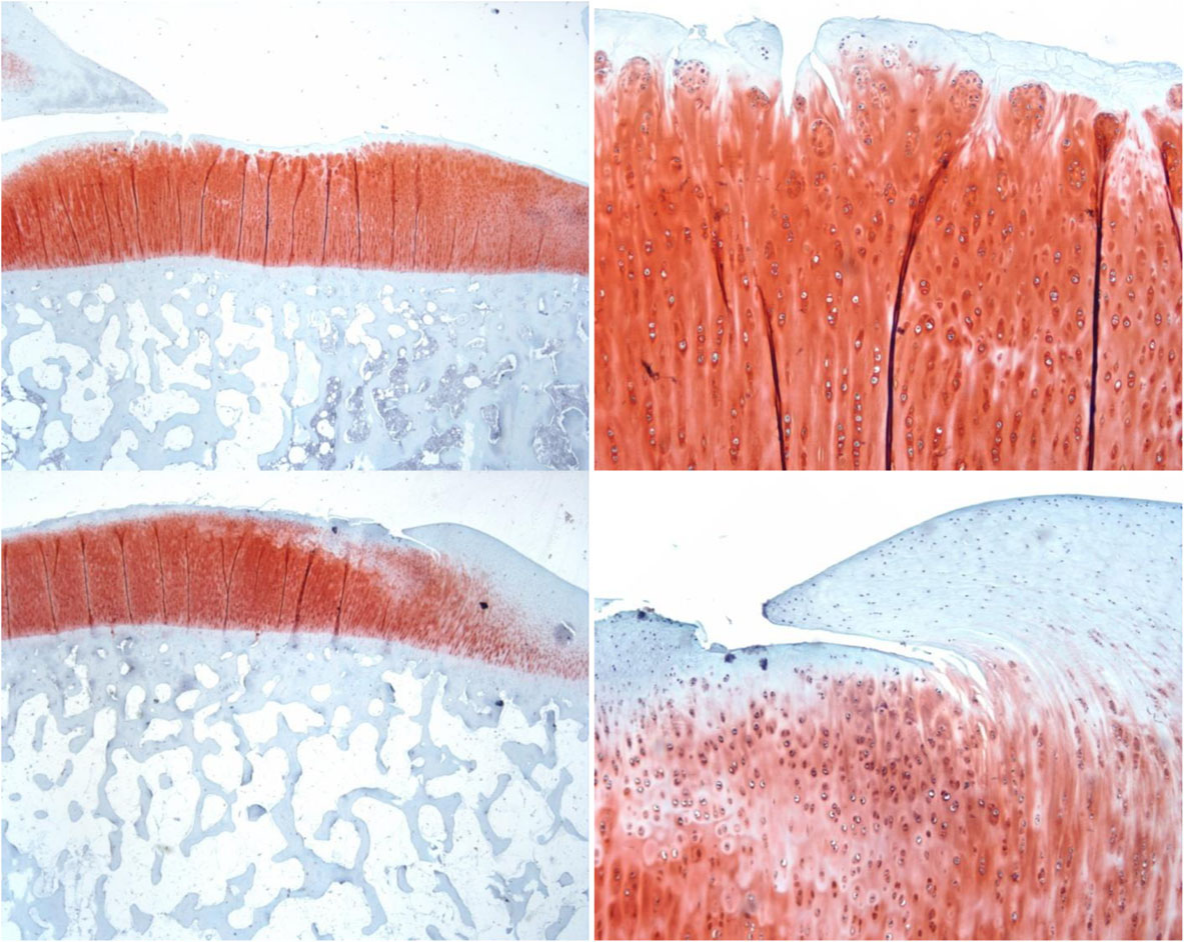
Tibia cartilage surfaces articulating against pyrolytic carbon (*top*) and Co-Cr (*bottom*), 52 weeks. ×2 (*left*), ×10 (*right*)

**Fig. 10:**
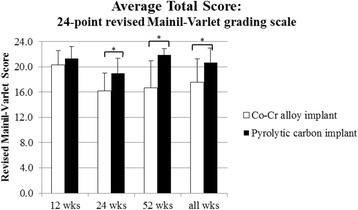
Mean total histologic scores for the medial menisco-tibial surface. *Significance, *p* < 0.05

Overall, the pyrolytic carbon implant group had superior histologic appearances for the medial tibia articulating surface over that of the Co-Cr alloy implant group at all evaluation periods (Fig. 9). In most pyrolytic carbon sections, the cartilage surface appeared normal with minimal mechanical damage or cellular changes. Isolated areas of cell clusters with early mechanical changes were observed in limited sections (Fig. 9, top right). Loss of matrix staining was observed for most Co-Cr alloy sections with cellular changes present, as well as mechanical damage at the cartilage surface (Fig. 9, bottom right). There was no significant difference in scores at 12 weeks; however, mean total histologic scores of the tibial articular cartilage were significantly improved at 24 weeks (*p* = 0.001) and 52 weeks (*p* < 0.001) for the pyrolytic carbon implant group (Fig. 10). Regarding within-animal paired comparison at 52 weeks, pyrolytic carbon was superior to Co-Cr alloy in all subcategories (*p* < 0.05). In eight of nine paired comparisons, the overall histologic score of the tibial surface was superior for the pyrolytic carbon implant side than the Co-Cr alloy side, with no statistically significant difference in the remaining comparison pair.

At 12 weeks, changes were primarily observed in the cellular morphology of the tibial cartilage, while structural changes were noted at both 12 and 52 weeks. Occasional loss of columnar orientation with cell clusters and loss of cell viability were observed at 12 weeks, as well as some loss of matrix GAG staining. At 24 weeks, cartilage surface changes were first observed with moderate irregularities, particularly for the Co-Cr alloy implants. Degenerative changes occurred to a lesser extent in the pyrolytic carbon group. Histologic scores decreased significantly from 12 to 24 weeks for both implant materials, with a greater decrease for the Co-Cr alloy group from 20.3 ± 2.3 to 16.2 ± 2.8 (*p* < 0.0001) than for the pyrolytic carbon group which decreased from 21.3 ± 1.9 to 19.0 ± 2.4 (*p* = 0.0005). Mean total histologic scores stabilized at 52 weeks compared to the earlier evaluation periods. The mean total histologic score of the pyrolytic carbon implants was significantly improved compared to that of the Co-Cr alloy specimens (*p* < 0.001) with mean total histologic scores of 21.9 ± 1.0 and 16.7 ± 4.3, respectively. Histologic appearances of the tibial cartilage and meniscus at 52 weeks were similar to those at 24 weeks for each respective implant material.

## Discussion

This study demonstrated that a pyrolytic carbon hemiarthroplasty implant material was superior to identical Co-Cr alloy implants. Less cartilage degeneration was observed in the cartilage adjacent to and in the vicinity of the pyrolytic carbon implants compared to the Co-Cr alloy implants in the medial femoral condyle at all evaluation periods. Surface cartilage wear, degradation, and cellular change were reduced when articulating against the pyrolytic carbon implants. In particular, changes in the tibial cartilage observed for the pyrolytic carbon group at 52 weeks were seen as early as 12 weeks for the Co-Cr alloy group. Both implant groups achieved a similar degree of direct bone-implant apposition at 12, 24, and 52 weeks.

The concept of a minimally invasive hemiarthroplasty for the treatment of focal osteochondral knee defects offers the advantages of reduced pain, a shorter hospital stay, and increased range of motion. Hemiarthroplasty also preserves bone stock, allowing for future revision or total joint replacement, if needed. While most devices require replacement of the opposing tibial articular surface, the device in this study does not and is comparable in concept to the current marketed device that requires resurfacing of only one articular surface—the HemiCAP (Arthrosurface Inc., Franklin, MA).

The HemiCAP consists of a bone fixation element and an articular component connected by a Morse taper. The bearing surface is Co-Cr-Mo alloy with titanium plasma spray on the underside for bone ongrowth. The biological and functional response of the HemiCAP was investigated in goats [16]. Radiologically, there was no evidence of implant failure or loosening nor any gross degenerative changes. Histological analysis showed new trabecular bone abutting the implant in all specimens at 1 year postoperatively. The medial femoral condyle cartilage lateral to the implant was fibrocartilage-like, whereas it was hyaline cartilage-like medial to the implant [16]. Histologic examination of the proximal tibia cartilage surface opposing the HemiCAP implant was characterized by the presence of fibrillations, similar to those observed in this study for the Co-Cr alloy group. Given its high fatigue and high wear resistance, biocompatibility, reduced elastic modulus, and propensity for SAPL adhesion to form a boundary lubricant, pyrolytic carbon offers the potential for improved clinical performance of implants for this partial joint resurfacing application compared to Co-Cr alloy.

One of the limitations of the surgical model used is that the implants were placed in a joint that was not truly arthritic. The implants were placed in a joint at the same time that the defect was made. This study also did not include control groups with osteochondral defects that were left untreated or, as previously stated, defects that were left untreated for a substantial period of time prior to implantation. It is important to note that it has been shown that untreated osteochondral defects result in deterioration of both the adjacent cartilage and the opposing cartilage on which it articulates [15].

In a study comparing the results of untreated critical-size cartilage defects in the medial femoral condyle of goats with defects treated with oxidized zirconium or Co-Cr small hemiarthroplasty implants after 52 weeks, 13 of 16 analyzed implants showed good osseointegration and high bone-implant contact (40–60%) [39]. Previous rabbit studies showed similar bone-implant contact percentages, ranging from 36 ± 4% [40] to 47.5 ± 4.7% [41]. The present study had comparable bone-implant contact with a mean of 34.5  ± 15.9% for the Co-Cr alloy implants and 30.2  ± 20.4% for the pyrolytic carbon implants.

Although fibrillation was rarely seen by Custers et al. [39], a previous study involving fixation of pyrolytic carbon and titanium implants in mongrel dogs showed moderate fibrillation of the opposing cartilage surface by the pyrolytic carbon implant and severe fibrillation by the titanium device at 12 months [42]. In the present study, no changes on the medial tibia plateau surface opposing the pyrolytic carbon implant were observed, but moderate fibrillation of the opposing tibia cartilage surface was observed for one of the three knees treated with Co-Cr alloy implants at 52 weeks. In another study by Cook et al. [28] using hip endoprosthesis in a canine model, areas of wear were much less extensive in animals with pyrolytic carbon devices, and there was significantly less fibrillation of opposing cartilage seen with the pyrolytic carbon implants compared to both Ti-6Al-4V and Co-Cr-Mo alloy devices (all *p* < 0.0075). Based on the direct comparison of pyrolytic carbon to identical metal alloy implants, it is hypothesized that the improved performance of pyrolytic carbon devices results from reduced material elastic modulus, as well as lower surface energy and the non-adhesive nature of the pyrolytic carbon surface [43]. In addition, the high affinity of SAPL to the surface of pyrolytic carbon assists in the formation of a boundary lubricant for reduced wear and friction during articulation [34, 35] which could contribute to the improved outcomes on the opposing cartilage surface.

Another limitation to this study is the small number of animals, which may skew statistical elements. However, bilateral surgery allows for intra-animal pair-wise comparison and therefore a reduced number of animals needed for the study. It also allows a direct comparison of the two implant materials. Studies performed in a larger sheep model comparing pyrolytic carbon implants to a chondroplasty control group are underway.

## Conclusions

In summary, the use of pyrolytic carbon as a hemiarthroplasty implant material was shown to be superior to Co-Cr alloy, which is the material that is currently being used in a marketed clinically available hemiarthroplasty knee device. Less degeneration was observed in the cartilage adjacent to and in the vicinity of the pyrolytic carbon implants compared to the Co-Cr alloy implants in the medial femoral condyle at all evaluation periods out to 1 year. There was also less wear, degradation, and cellular changes of the tibial cartilage surface when articulating against the pyrolytic carbon implants. Changes in the tibial cartilage observed for the pyrolytic carbon group at 52 weeks were seen as early as 12 weeks for the Co-Cr alloy group. Again, larger sheep models comparing a clinical pyrolytic carbon device to control chondroplasty-treated defects are underway.

## Abbreviations

ANOVA: Analysis of variance
AP: Anterior-posterior
Co-Cr: Cobalt-chromium
Co-Cr-Mo: Cobalt-chromium-molybdenum
DPPC: Dipalmitoylphosphatidylcholine
MFC: Medial femoral condyle
SAPL: Surface-active phospholipids
TKA: Total knee arthroplasty
